# The Next Generation of Molecular and Cellular Therapeutics for Inherited Retinal Disease

**DOI:** 10.3390/ijms222111542

**Published:** 2021-10-26

**Authors:** Luis A. Martinez Velazquez, Brian G. Ballios

**Affiliations:** 1 Department of Ophthalmology, Massachusetts Eye and Ear, Harvard Medical School, Boston, MA 02114, USA; luis_martinezvelazquez@meei.harvard.edu; 2 Department of Ophthalmology and Vision Sciences, Temerty Faculty of Medicine, University of Toronto, Toronto, ON M5T 3A9, Canada

**Keywords:** CRISPR, gene editing, gene therapy, inherited retinal disease, retina, neuroprotection, antioxidant, stem cells, optogenetics, RNA modification, AONs

## Abstract

Inherited retinal degenerations (IRDs) are a diverse group of conditions that are often characterized by the loss of photoreceptors and blindness. Recent innovations in molecular biology and genomics have allowed us to identify the causative defects behind these dystrophies and to design therapeutics that target specific mechanisms of retinal disease. Recently, the FDA approved the first in vivo gene therapy for one of these hereditary blinding conditions. Current clinical trials are exploring new therapies that could provide treatment for a growing number of retinal dystrophies. While the field has had early success with gene augmentation strategies for treating retinal disease based on loss-of-function mutations, many novel approaches hold the promise of offering therapies that span the full spectrum of causative mutations and mechanisms. Here, we provide a comprehensive review of the approaches currently in development including a discussion of retinal neuroprotection, gene therapies (gene augmentation, gene editing, RNA modification, optogenetics), and regenerative stem or precursor cell-based therapies. Our review focuses on technologies that are being developed for clinical translation or are in active clinical trials and discusses the advantages and limitations for each approach.

## 1. Introduction 

Inherited retinal degenerations (IRDs) are a genetically and clinically heterogeneous group of disorders that are often characterized by severe loss of vision. While these disorders have long been considered untreatable, recent advances in molecular biology have led to the first FDA-approved gene therapy for a retinal dystrophy, voretigene neparvovec-rzyl (LUXTURNA) for *RPE65*-associated Leber congenital amaurosis (LCA). Currently, more therapies for other disorders are being evaluated in over 60 ongoing interventional clinical trials (clinicaltrials.gov) and preclinical studies. Many therapies currently in development are designed to treat IRDs associated with a specific genotype; nevertheless, the field is increasingly focused on generalizable treatment strategies that can be applicable to a broad range of retinal dystrophies and degenerations. Here, we will review multiple strategies that are finding application in clinical investigation for the treatment of IRDs with a focus on neuroprotection, gene augmentation, gene editing, optogenetics, and stem and/or precursor cell therapies. 

Vision science researchers have been at the forefront of innovations in gene therapy and regenerative medicine. This is not a coincidence, given that the eye offers many advantages for the study and development of these technologies. There are structural advantages to these studies in the eye, including its organization into multiple compartments that enable accurate localized delivery of cell and molecular therapies, such as viral vectors, near the intended target tissue and under direct visualization [1]. The eye has long been considered an immune-privileged organ, potentially reducing the inflammatory response associated with the delivery of gene therapies [2,3,4], and while antibodies against viral vectors have been reported following therapy of one eye [5], treatment of the second eye has been shown to be safe and successful [6]. Nevertheless, monitoring the eye for inflammation after viral-mediated gene therapy remains an important consideration. The fellow eye can also serve as the untreated control in clinical trials to determine the efficacy of the therapy being evaluated, which is crucial given the heterogeneity that can be present in disease progression in retinal dystrophies between individuals [3]. Additionally, there is a long list of non-invasive methods to test the visual system with objective functional (electroretinography, ERG) and structural (retinal imaging) metrics, as well as psychophysical testing (visual fields, microperimetry), to evaluate changes after therapy. Some of the advanced, multimodal imaging technologies include optical coherence tomography (OCT), fundus autofluorescence, and color fundus photography. However, much of the progress in clinical therapies would have been impossible without the existence of a variety of preclinical animal models for inherited retinal degenerations, which have fueled the rapid growth in our understanding of the pathobiology of vision loss. These models have allowed for the generation of innovative experimental therapies and proof-of-principle experiments that demonstrated their therapeutic benefit in slowing down retinal degeneration and even restoring vision. 

## 2. Genomics of Inherited Retinal Disease

Advances in molecular genetics over the past 30 years have led us to identify mutations in more than 300 different genes as causative for heritable retinal degenerations [7,8]. While some of the identified mutations are relatively rare, a recent study calculated that approximately 2.7 billion people worldwide, or more than one- third of humans, are healthy carriers of a mutation in a gene associated with an autosomal recessive IRD [7]. This is perhaps the highest rate of any group of Mendelian conditions in humans. Indeed, most of these hereditary retinal dystrophies are monogenic (single-gene) disorders that follow classic Mendelian inheritance patterns such as autosomal dominant, autosomal recessive, and X-linked transmission [3]. In large part due to innovations in next-generation sequencing (NGS) technologies [9], clinical genetic testing is increasingly available and more affordable, allowing for many of the patients diagnosed with a clinical retinal dystrophy to achieve a molecular diagnosis [10]. 

Current estimates suggest that 90% of patients with an IRD have a mutation in one of the already known causative genes [11]. To decrease cost and improve genetic testing access, retinal dystrophy gene panels and clinical syndrome-specific gene panels (e.g., for macular dystrophies) have been generated [12] and are used in academic medical centers and clinical laboratories. Through these approaches, the causative mutation can be identified in approximately 70% of patients [13], with some variability depending on the clinical syndrome or population tested. For those patients in whom the causative mutation is not identified through these methods, whole exome and whole genome sequencing can be performed, and it will hopefully become more routine in the future as the cost and availability for genetic sequencing continues to improve [12]. The field of ophthalmic genetics also benefits from genetic sequencing data- sharing networks through global databases such as ClinVar [14], and the Leiden Open Variation Database [15]. Through more patient sequencing and genetic analysis, we will identify more genes involved in retinal dystrophies, and recognize complex variants that affect coding and noncoding regulatory regions causing retinal dystrophies. 

With the increased availability and sensitivity of genetic sequencing technologies, some IRD specialists have advocated for the sequencing of all patients and the replacement of the historical clinical diagnostic names for syndromes with a molecular-based nomenclature for the conditions [16]. This proposal acknowledges the genetic heterogeneity of clinical IRD syndromes, including retinitis pigmentosa (RP) (caused by mutations in over 75 genes) [8] and Leber Congenital Amaurosis (LCA) (caused by mutations in over 24 genes) [17], and it emphasizes the importance of mutation identification. This is perhaps even more relevant when practicing at a time where more and more gene therapies are in development. 

Another advantage of widely available genetic testing is that it has helped us estimate the prevalence of different causative gene mutations and specific alleles, helping us better direct resources for the development of future therapies. While the allele frequency depends on the studied population, in aggregate, mutations in the ABCA4 gene (Stargardt disease), EYS (associated with non-syndromic RP, cone-rod dystrophy, and LCA), USH2A (associated with Usher syndrome, type 2, as well as non-syndromic RP), CEP290 (associated with LCA, type 10), and MYO7A (associated with Usher syndrome, type 1) account for more than one third of all cases of IRDs [7,18,19,20]. Not surprisingly, these genes have become prime candidates for the generation of new gene therapies. However, treatments of prevalent variants in each of these genes pose unique molecular challenges. Table 1 summarizes many of the recent clinical trials for inherited retinal disease, and these strategies will be explored in further detail in this review. 

## 3. Neuroprotection

The common feature that unites retinal dystrophies is the early loss of photoreceptor cells. These are the light-responsive cells of the retina and consist of the high-sensitivity rods and the cones, which are activated by bright light of different colors depending on which photopigment they express. Cones are concentrated in the macula, while rods are present throughout the macula and peripheral retina. Photoreceptors are terminally differentiated neurons that are not regenerated after degeneration or injury in adult mammals [38]. Most of the autosomal recessive dystrophies are caused by mutations in genes expressed by photoreceptors and retinal pigmented epithelium cells (RPE) that are necessary for the normal development, function, and survival of photoreceptor cells [8,38,39]. Given the long list of mutations that can be responsible for IRDs and the high prevalence in the population of acquired retinal degenerations, such as age-related macular degeneration (AMD) and diabetic retinopathy, there is a desire to develop treatment methods agnostic to the molecular mechanism of the underlying condition. 

### 3.1. N-Acetylcysteine and Antioxidant Therapies for Neuroprotection 

Neuroprotection may broadly encompass any potential treatment that provides support to neurons to prevent them from undergoing degeneration as a result of the ongoing disease process. While there are no current FDA-approved neuroprotective therapies for retinal disorders, a long list of candidate molecules and strategies have been evaluated for their potential role in delaying photoreceptor degeneration and are reviewed elsewhere [40,41,42]. One strategy currently being evaluated in clinical trials is the use of antioxidants as neuroprotective agents. Preclinical models have implicated a role for oxidative damage in photoreceptor degeneration [43,44], and N-acetylcysteine and other antioxidants [45] have shown pre-clinical promise as potential drugs to slow down retinal degeneration in RP [46]. 

N-acetylcysteine (NAC) is widely used clinically as a mucolytic agent [47], and as an antidote for acetaminophen toxicity [48]. NAC is a liposoluble molecule that can readily permeate cell membranes, where it serves as a precursor of cysteine and stimulates the formation of glutathione, a powerful antioxidant, in neurons [49]. Glutathione (GSH) is a tripeptide of glutamate, cysteine, and glycine that offers protection against oxidative stress in the retina [50]. Oxidative stress has been proposed as a key player in neurodegeneration in a wide range of ocular disorders including glaucoma, the acquired age-related macular degeneration (AMD), and inherited retinal dystrophies such as RP [50]. In animal models of typical RP and in human disease, rods are the first cells to degenerate, and cone cell death does not begin until most rods have degenerated [44]. There are several hypotheses as to why mutations that cause rod photoreceptor degeneration invariably lead to the gradual dysfunction and death of cone photoreceptors, including a role for oxidative stress. In healthy retinas, rods account for 95% of the cells in the outer retina, and in their absence, oxygen consumption by the outer retina is reduced. This leads to higher concentrations of oxygen experienced by other cells in the retina [45,51,52]. A Phase 1, open-label study performed at Wilmer Eye Institute tested the safety of oral dosing of NAC for patients with retinitis pigmentosa (Fight RP, NCT03063021). The results of this study suggested a steady visual improvement over the course of the 24-week dosing period [53], and they have motivated an extension study (NCT03999021) to determine if the visual gains become clinically significant over a longer dosing period. 

### 3.2. Pharmacological and Neuroprotective Strategies for the Treatment of Stargardt Disease

Stargardt disease is the most common inherited macular dystrophy and leads to vision loss at a young age. Mutations in the ABCA4 retinal transporter protein result in toxic accumulation of by-products from the vitamin A cycle on the photoreceptor disc membranes. These discs are phagocystosed by RPE cells and result in the production of toxic bis-retinoids such as N-retinylidene-N-retinylethanolamine (A2E) and lipofuscin, ultimately leading to RPE atrophy, photoreceptor degeneration, and permanent vision loss [54,55]. Multiple clinical trials are targeting different steps in the vitamin A cycle as a potential therapy for Stargardt disease [56]. ALK-001, a deuterated form of vitamin A (C20-D3-vitamin A), was shown in a murine model to slow down the rate of vitamin A dimerization without affecting retina function [57] and is currently being evaluated in a Phase 2 clinical trial (NCT02402660, Alkeus Pharmaceuticals). Fenretinide, LBS-008, STG001 (NCT04489511, Stargazer Pharmaceuticals), and A1120 are other novel therapeutics that have been shown in preclinical models to selectively deplete vitamin A from the eye through competitive inhibitory mechanisms on retinal binding protein-4 (RBP-4) [56,58]. Emixustat is a small molecule that targets the RPE65 enzyme to reduce the production of the visual chromophores (11-cis-retinal) from vitamin A, thereby reducing the accumulation of toxic A2E and lipofuscin [54]. Emixustat is currently being evaluated in a Phase 3 clinical trial for the treatment of Stargardt disease (NCT03772665, SeaSTAR, Kubota Vision). Finally, soraprazan/remofuscin is a small molecule that was shown to increase the removal of existing lipofuscin from the RPE after delivery through intravitreal injection in Abca4 mutant mice [59]. 

Age-related macular degeneration (AMD) accounts for 8.7% of cases of blindness and affects millions of individuals worldwide [60]. AMD is an acquired retinal degeneration, although numerous studies have identified genetic variants in CFH, CFI, C2, C3, C9, and ARMS2/HTRA1 [61,62], which may confer increased risk to carriers. Genetic linkage studies suggest that 50% of eyes with dry AMD and geographic atrophy (GA) have polymorphisms in complement regulatory proteins when compared to age-matched controls [63]. AMD is generally sub-classified into non-exudative AMD or exudative AMD, depending on whether disease has progressed to choroidal neovascularization (CNV). While vascular endothelial growth factor inhibitors (i.e., anti-VEGF medications) have become the mainstay of treatment for exudative AMD, there are no approved therapies for non-exudative AMD [64] except for the combination of nutritional supplements demonstrated in the Age-Related Eye Disease Studies (AREDS [65] and AREDS2 [66]). These were shown to delay the progression of AMD in patients with intermediate or late non-exudative AMD. 

The complement pathway, an essential part of the innate immune system, has been suggested as a potential common pathway between Stargardt disease and AMD [67,68]. Avacincaptad pegol (Zimura, Iveric Bio), which targets complement factor C5, was shown to be effective in reducing the progression of GA secondary to AMD in a Phase 2/3 trial (GATHER1, NCT02686658). In GATHER1, patients with AMD were monitored for the progression of GA through fundus autofluoresence (FAF) at three timepoints: baseline, six months, and twelve months, among patients that received one of two doses of avacincaptad pegol through intravitreal injection (2 mg, 4 mg) versus placebo. Both doses of the treatment resulted in statistically significant reduction in the progression of GA (27.4% for the 2 mg dose and 27.8% for the 4 mg dose) when compared to placebo [63]. Avacincaptad pegol is now undergoing a Phase 2b trial (NCT03364153) to test its efficacy in delaying progression of Stargardt disease. 

### 3.3. Neurotrophic Factors and Stem-Cell Based Therapies for Neuroprotection

Preclinical work has shown that neurotrophic factors such as ciliary neurotrophic factor (CNTF) [69] and glial-derived neurotrophic factor (GDNF) [70,71] can slow photoreceptor degeneration in several IRD animal models. These studies inspired clinical trials for the treatment of patients with RP with an implant designed for the gradual release of CNTF; unfortunately, this study did not demonstrate improvement in visual acuity, visual field sensitivity, or retinal structure in treated subjects [72]. Prior studies have suggested that a broad set of neuroprotective factors are secreted in healthy retina to support neuronal survival, and the combined administration of these trophic factors were shown to be more effective for the survival of retinal ganglion cells (RGCs) than the administration of each factor individually [73,74]. These studies led to the hypothesis that the sustained expression of multiple neurotrophic factors would be required to successfully delay retinal degeneration. Stem and progenitor cells are actively being studied as a possible source for these neurotrophic factors in preclinical studies and clinical trials. 

Bone marrow stromal cells (hBMSC) are multipotent stem cells that normally give rise to bone, cartilage, and mesenchymal cells (MSCs), which were shown to differentiate into multiple cell types including glial cells and neurons [75,76]. While their ability to differentiate into many tissues now seems limited [75], MSCs were found to secrete growth factors and cytokines that normally support hematopoietic progenitor cell proliferation and differentiation. This includes VEGF and bFGF [77], as well as neurotrophic factors such as brain-derived neurotrophic factor (BDNF), nerve growth factor (NGF), GDNF, and CNTF [75,76,78,79,80]. Prior to the work suggesting MSCs’ therapeutic potential for the retina, neurotrophic factors secreted by MSCs had been implicated in promoting neuronal survival and improved function after brain injury. A study performed in an animal model of ischemic cerebral injury showed that transplanted human BMSCs decreased apoptosis at the ischemic boundary zone and provided trophic factors such as BDNF and NGF [81]. Importantly, the study also reported that only a small subset of the transplanted BMSCs (1–5%) expressed proteins found in mature parenchymal neurons [81], suggesting that neurotrophic support by the MSCs was the primary mechanism of improved function and neuronal survival, rather than the incorporation of newly differentiated neurons [81]. 

Yu et al. transplanted BMSCs in an animal model of glaucoma and found that these also promoted the survival of RGCs and expressed neurotrophic factors such as CNTF, GDNF, and BDNF in the retina [79]. This hypothesis was further supported by evidence that in other models of optic nerve injury, including glaucoma, optic nerve crush, and transection, the degeneration of RGCs and their axons can be delayed by the transplantation of BMSCs to the vitreous [76]. Subsequent work also demonstrated that MSCs could be pre-differentiated into glial-like cells prior to transplantation, which can further provide neurotrophic support to injured neurons [82]. 

The therapeutic potential of neurotrophic factors provided by MSCs was also tested in other models of retinal injury and IRD. For example, in an ischemic retina rodent model, MSCs injected into the vitreous cavity were shown to secrete CNTF, bFGF, and BDNF, and they yielded a small number of newly differentiated neurons [78]. These findings reinforced that neuroprotection is the primary mechanism of therapeutic benefit with MSC transplantation. BMSC-conditioned media was also shown to delay photoreceptor cell apoptosis in an in vitro model using a mouse retinal cell culture [83]. This same study also found that by injecting MSCs into the subretinal space of the Royal College of Surgeons (RCS) rat model of retinal degeneration, which is caused by mutations in the receptor tyrosine kinase gene MerTK [84], retinal function was preserved, and histological findings of degeneration were delayed [83]. In a different study using the RCS rat model, BMSCs administered intravenously were shown to prevent photoreceptor loss and preserve visual function; again, it was suggested that this was through the expression of neurotrophic factors including CNTF [80]. MSCs from other sources including those derived from umbilical cord blood have been shown to be neuroprotective of ganglion cells [80], and other sources, such as dental pulp derived MSCs [76], are active areas of study. 

Multiple groups have used MSCs from diverse sources to test their neuroprotective role in preclinical animal studies and Phase 1/2a trials that are reviewed in further detail elsewhere [42,85]. Of note, umbilical cord-derived stem cells (hUTCs) (NCT02895815) and fetal brain-derived neural progenitor stem cells (NCT01632527) have been explored in therapies for dry AMD, and although early-stage trials supported the safety of these therapies, both studies were terminated [42], and some of the reported concerns regarding hUTCs transplantation include epiretinal membrane formation. Histologic examination of the surgically-excised membranes from patients revealed that these are made up of a combination of host cells and donor cells [86]. 

Taken together, these studies support the hypothesis that a combination of neurotrophic factors could be effective in delaying neuronal death in the degenerating retina, and that stem cells or progenitor cells could provide a sustained source for these factors, which might be necessary to see a therapeutic benefit. Retinal progenitor cells (hRPCs) isolated from fetal human eyes, are currently being investigated as treatment for retinitis pigmentosa (RP). jCyte, a California-based company, is testing the neuroprotective effects of hRPCs delivered with intravitreal injections (clinicaltrials.gov identifier NCT03073733). Like studies with MSCs, jCyte aims to demonstrate that hRPCs can provide neurotrophic support restoring function in the remaining photoreceptor cells and preventing their rapid degeneration. In addition to providing neurotrophic factors, hRPCs have been shown in preclinical models to differentiate into photoreceptors and other retinal cell-types at low efficiencies, which might be able to replace the photoreceptors and other retinal cell-types that have been lost in disease [42]. The role of cell replacement therapeutics will be further discussed later in this review. 

jCyte recently announced the results of the Phase 2b trial for their jCell product at the American Society of Retina Specialists annual meeting in 2020. This trial met its primary endpoint, demonstrating an early and sustained improvement in the best corrected visual acuity (BCVA) in patients treated with a single dose of 6-million hRPCs when compared to patients that received placebo [87]. These patients also demonstrated positive results in secondary visual function endpoints including improvements in peripheral visual field area, contrast sensitivity, and better ambulation in low light settings measured by the low light mobility test (LLMT) [87]. Patients that received a lower dose of 3-million hRPCs failed to demonstrate meaningful improvement [87]. 

## 4. Gene Replacement Therapies: Early Successes and Future Challenges

Advancements in molecular biology and a detailed understanding of the pathophysiology of retinal disease allowed for the development of the first FDA-approved gene therapy for biallelic RPE65-associated Leber congenital amaurosis (LCA), voretigene neparvovec-rzyl (LUXTURNA). RPE65 is an isomerase required for the visual pigment cycle that acts in retinal pigment epithelium (RPE) cells. Before gene therapies for retinal disease could be developed, researchers had established animal models in multiple species including rodents [88,89] and the Briard dogs [90,91] that led to the understanding of the physiologic role of the RPE65 enzyme in visual function and disease. Then, these models formed the basis for the development and validation of experimental gene therapies designed to replace the function of mutated RPE65 [90,91,92]. While small animal models were beneficial in the lab to study the cell biology of disease, due to the ease of genetic manipulation and their shorter life cycles, larger animal models were necessary for the preclinical development of surgical strategies for vector delivery relevant to humans. Simultaneously, natural history studies of disease progression in RPE65-associated retinal dystrophy demonstrated that these patients had slower anatomic progression with respect to photoreceptor cell loss than other forms of LCA [93,94,95]. This significant structure-function dissociation provides a setting for sufficient cells to serve as a substrate for gene replacement. Three independent clinical trials were conducted using different vectors based on the adeno-associated virus (AAV)-2 to deliver a healthy copy of the RPE65 coding sequence in trial subjects [96,97,98]. 

An important lesson learned from the successful RPE65 gene therapy studies is the need for innovation in measuring improvement in visual function in a patient population with low vision [16]. Beyond central visual acuity, outcome metrics needed to encompass both physiologically relevant responses, as well as responses meaningful to the limitations that patients experience in their activities of daily living. For example, some of the metrics that were applied during the RPE65 trials included pupillometry [99], full-field sensitivity testing [100], microperimetry [101], dark-adapted sensitivity [102], and visual mobility testing [6,96,98,100,103]. The latter test, also known as the multi-luminance mobility test (MLMT), was developed and validated specifically for these studies, and it was able to assess the functional challenges these patients experience with low-luminance vision [104]. MLMT was an important outcome for determining treatment response during the Phase 3 efficacy trial for voretigene neparvovec-rzyl [105]. Variants of this assay are now being used in other interventional trials for IRDs. 

The success of voretigene neparvovec-rzyl has sparked a search to identify other retinal dystrophies that can also be treated through gene augmentation: that is, the use of a viral vector to deliver a normal copy of the affected gene. This strategy can be applied for the treatment of loss-of-function mutations, and many current Phase 1/2 clinical trials are using this approach for specific IRDs [16,106]. As a gene delivery vector, AAVs have been the virus of choice for IRD therapy efforts over the past 30 years [3]. In particular, the AAV2 and AAV8 vectors have demonstrated success in the delivery of genetic material to photoreceptor cells in the outer retina. Vectors are engineered to deliver a normal copy of the gene of interest to the photoreceptors or retinal pigment epithelium (RPE) cells and are generally delivered surgically via subretinal injection or in some cases through intravitreal injection [106]. An AAV vector was first reported to be successful for the treatment of a preclinical model of IRD in 1998, when it was used to restore visual function in the retinal degeneration slow (rds) mouse through the reintroduction of a wild-type copy of the PRPH2 gene [107]. 

Other clinical trials exploring the use of AAV vectors for the treatment of other IRD targets are summarized in Table 1. The number of concurrent sponsored studies across a variety of IRDs reflects the promise of gene therapy approaches for disease. Several, though not all, of these early trials are showing promising results, and moving to more advanced stages. Current studies include Phase 1/2 trials in CNGA3- [108] and CNGB3-associated achromatopsia (Sponsors: AGTC; MeiraGTx; STZ eyetrial). The results for one of the Phase 1 trials for the delivery of CNGA3 using an AAV8 vector (NCT02610582, Sponsor: STZ Eyetrial) were published in 2020, and the results demonstrated the safety of the therapy and improvement in contrast sensitivity (mean 0.33 log) and improvement in visual acuity (mean 2.9 letters) among its nine participants [108]. Active Phase 1/2 trials are also underway for RS1-mediated X-linked retinoschisis (Sponsors: AGTC; National Eye Institute, NEI). Unfortunately, results for the NEI’s Phase 1 studies using an AAV8 vector to deliver RS1 (NCT02317887; Sponsor: NIH/NEI) failed to demonstrate a significant improvement on visual acuity, retinal sensitivity, or electroretinography (ERG). Adverse events associated with the therapy included intraocular inflammation in four out of the nine participants [109]. 

Phase 1/2 (Sponsors: AGTC; MeiraGTx; 4D Molecular Therapeutics) and Phase 2/3 trials (Sponsor: Biogen/NightstaRx Therapeutics) are also underway for RPGR-associated X-linked retinitis pigmentosa (XLRP). Results for the Phase 1/2 trial using an AAV8 vector to deliver a codon-optimized copy of RPGR (NCT03116113, Sponsor: Biogen/NightstaRx) were published in 2020, and they demonstrated safety for this therapy, aside from mild subretinal inflammation (at the site of the injection) seen in patients that received higher doses of the therapy (up to 5 × 1012 genomic particles (gp)/mL) that responded to oral steroids [110]. The study also demonstrated an improvement in retinal sensitivity and reversal of visual field loss in seven out of the 18 patients (doses of 5 × 1011 up to 5 × 1012 gp/mL) that lasted throughout the 6-month follow-up period [110]. However, it was announced in May, 2021 that the subsequent Biogen-sponsored Phase 2/3 study failed to meet primary endpoints of ≥7 dB improvement from baseline in ≥5 of the 16 central loci in the 10-2 grid assessed by microperimetry at 12 -months post-treatment. Nevertheless, positive trends were reported across some secondary clinical endpoints. Meanwhile, the MeiraGTx-sponsored Phase 1/2 study for RPGR-associated XLRP showed that the AAV5-RPGR product not only met safety endpoints, but also showed meaningful improvements in secondary functional endpoints at three months, with sustained or improved effects at six months follow-up for the low and intermediate doses. These findings were assessed with static perimetry and microperimetry, and significant differences were found between treated and untreated eyes in mean retinal sensitivity, central visual field progression. MeiraGTx is now moving to a sponsored Phase 3 study for this vector. 

Other important RP trials include a Phase 1/2 trial in PDE6B-associated autosomal recessive (ar) RP (Sponsor: Horama S.A.), a Phase 1/2 trial in RLBP1-associated arRP (Sponsor: Novartis), and a Phase 1/2 trial in MERTK-associated arRP (Sponsor: King Khaled Eye Hospital). The results for the Phase 1 trial using an AAV2 vector to deliver MERTK (NCT01482195, Sponsor: King Khaled Eye Specialist Hospital) demonstrated safety of the subretinal delivery of this vector based on a two-year follow-up period. The investigators also reported improved visual acuity in the treated eye in three out of the six eyes that received the therapy, but the gains were lost within two years in two of the treated eyes [111]. The results of a Phase 1 trial using an AAV5 vector to deliver GUCY2D in patients with LCA1 (NCT03920007; Sponsor: Atsena Therapeutics) are also available and demonstrated safety in the use of this treatment in one eye of three patients over a nine-month follow-up period with evidence of improvement in vision [112]. Two patients demonstrated improvement in rod photoreceptor function as measured by full-field stimulus testing, one patient had improved pupillary responses, and one patient had gains in BCVA of 0.3 logMAR [112]. Finally, there are also ongoing Phase 1 and Phase 2 trials in CHM-associated choroideremia (Sponsors: 4D Molecular Therapeutics; Spark Therapeutics; STZ Eyetrial; University of Oxford; Bascom Palmer/U of Miami; University of Alberta; Biogen/Nightstar Therapeutics). Of note, Biogen recently announced the top-line results of its Phase 3 gene therapy study in choroideremia (STAR study, NCT03496012) for its timrepigene emparvovec (BIIB111/AAV2-REP1). The study did not meet its primary endpoint of fraction of participants that demonstrated greater than a 15-letter improvement from baseline BCVA by using the Early Treatment of Diabetic Retinopathy (ETDRS) chart at 12-months follow-up. The study also failed to demonstrate efficacy on secondary endpoints; however, further long-term analysis is forthcoming. 

One of the major limitations of the AAV vector, in IRD gene therapy specifically, is its small size, which limits the delivered coding sequence to approximately 4.7-kilobases (kb) [113]. This has prevented its ready application in the treatment of common retinopathies caused by genes with longer coding sequences, such as the ABCA4 gene (6.8-kb), which is the most common cause of hereditary macular dystrophy. For this reason, other viral vectors such as lentiviruses, which can deliver coding sequences up to 8 -kb in length, have been developed with the hope they will facilitate the delivery of larger genetic payloads and stable integration into the genome of transduced cells. Nevertheless, lentiviruses have limitations of their own, including their poor efficiency at transfecting photoreceptor cells and the theoretical risk of tumorigenicity given their random integration of their coding sequences into the cells’ genome. A lentiviral strategy delivering a wild-type copy of ABCA4 for the treatment of Stargardt disease, as well as MYO7A for the treatment of Usher syndrome, type 1B, were studied in Phase 1/2 clinical trials, but these studies were discontinued by the sponsor (ABCA4, NCT01736592, NCT01367444; MYO7A, NCT01505062 Sanofi) [114] (Table 1). Further studies for these approaches have not yet been announced. 

Other alternatives that are being explored for the delivery of large genes to the retina include split-gene approaches, in which the coding sequence of a large gene is split and then packaged into separate vectors for delivery. Multiple laboratories have effectively used this split vector approach in preclinical models: first for the delivery of the erythropoietin genomic locus [115], but later for retinal disease genes caused by variants in the MYO7A gene (Usher syndrome, type 1B) [116,117] and the ABCA4 gene (Stargardt disease) [117]. This approach relies on the recombination of the two vectors in co-infected cells to generate the full-length coding sequence of the gene. It remains to be seen how this approach will perform in clinical studies. 

CEP290 is also a large gene (8-kb) that has drawn significant attention for gene-therapy approaches. Variants in CEP290 are the most frequent causes of LCA, accounting for more than 25% of clinical cases. The clinical disease has features of structure-function dissociation, like RPE65-associated disease, that make it an attractive candidate for gene augmentation therapy. This includes the prolonged preservation of the outer retinal structure in the fovea and central macula: the area of the retina responsible for the highest visual acuity. Researchers are pursuing approaches such as using a partial gene product, known as the “miniCEP290” fragment, which has been shown to restore function in a mouse model of CEP290-LCA, and it is small enough to be delivered in an AAV vector [118]. Another important ongoing clinical trial aims to use gene-editing technologies, for the first time in humans [119], to restore a wild-type copy of CEP290 in a patient’s photoreceptor cells [HH8] [120]. These technologies will be further described later in this review. 

The safety of AAV vectors in the eye is supported by the absence of major adverse events in follow-up studies, now surpassing 10 years for patients that received treatment for RPE65. Functional assays to detect the presence of neutralizing antibodies were performed as a part of these trials, which found that a subset of patients developed antibodies against the AAV2 capsid [5,96,97]. Of note, these responses were smaller than those recorded from individuals receiving systemically injected AAVs [121], which was likely due, in part, to the lower concentrations of virus required in the eye [122]. Similar studies used an enzyme-linked immunosorbent spot assay (ELISPOT) to demonstrate that antibodies were not generated against RPE65, with two exceptions that were considered to be artifactual [96,123]. Similar results were reported after the NEI sponsored X-linked retinoschisis trial in which ocular inflammation was reported as the main adverse event following treatment, and the patients’ sera tested positive for neutralizing antibodies against the AAV8 capsid but not the RS1 protein [109]. Of note, AAV delivery in this trial was intravitreal. Antibodies against AAV2 were also detected in the Phase 1 trial for MERTK-associated arRP [111]. The measurable humoral response to AAV capsids has raised the concern about the timing of treatment for the contralateral eye, and animal studies support that neutralizing antibody generated after treatment of the first eye could reduce the efficacy of treatment in the contralateral eye [124]. The extent to which antibodies are present might be dependent on the ocular compartment in which the AAV is delivered. While neutralizing antibodies have been detected after intravitreal injection in animal models [124] and in human trials [109], the sub-retinal compartment appears to be an immune-privileged site [125]. The eye possesses the ability for immune modulation with mechanisms such as the anterior chamber-associated immune deviation (ACAID), which rely on the induction of Tregs, anti-inflammatory M2 macrophages, and the generation of cytokines that promote immune tolerance [122]. A similar mechanism to ACAID has been suggested to account for the immune tolerance of the subretinal space in response to AAV therapy [126]. Nevertheless, localized inflammation was reported in patients that received subretinal injections with high doses of the AAV8-coRPGR construct (5 × 1012 gp/mL); however, the inflammation was responsive to oral steroids [110]. 

Finally, while the use of AAV vectors in the eye has led to minimal adverse effects, their use for systemic genetic disorders has proven more challenging. The administration of higher doses systemically was linked to liver failure, sepsis, and the death of two study participants in an Audentes Therapeutics’ Phase 2 gene therapy trial for X-linked myotubular myopathy [127]. It is important to note that none of the patients who received a lower dose of the therapy had liver-related adverse events. Other notable examples of high-dose systemic trials of AAV therapies (at least 2 × 1014 vector genomes (vg)/kg) with reported toxicities mainly related to immune responses include AveXis’s Zolgensma (onasemnogene abeparvovec) for spinal muscular atrophy, as well as Solid Biosciences’ SGT-001 and Pfizer’s PF-06939926 for Duchenne muscular dystrophy [128]. 

### 4.1. Improving Cell-Type Specificity and Delivery Efficiency of AAV Vectors

Different AAV serotypes have different tropisms for retinal cell types, and this characteristic may be used to achieve cell-type specificity of expression of the delivered construct. Scientists continue to modify viral capsids through point mutations with the hope of identifying AAV variants that can achieve higher expression levels and transduction efficiency in the cell-type of interest. This approach also holds promise at reducing immunogenicity by reducing the formation of neutralizing antibodies [129,130]. Avoiding immunogenicity is essential given that one eye is treated at a given time with gene therapy and the presence of neutralizing antibodies, which have been reported after intravitreal injection [124], would theoretically reduce the transfection efficiency and expression levels of the construct when treating the second eye. 

Before a new vector is ready for clinical testing, its expression must be confirmed in the correct cell-type and at appropriate levels to possibly restore visual function. Human retinal organoids, which are self-organizing retinal tissue differentiated from induced pluripotent stem cells (this topic will be further discussed in the cell replacement therapies section), now allow for the testing of vector tropisms in human photoreceptors [131,132]. This model has been used to screen for AAV capsid serotypes that have improved transduction efficiencies in the retina. This has yielded encouraging results with a specific AAV2 variant (7m8), which achieves up to 60% transduction efficiency [125,131,132,133]. While AAV2 has provided excellent success in transducing the outer retina, its efficiency is lower in the inner retina [134]. Targeted mutations of the capsid in multiple surface tyrosine (Y) residues to phenylalanine (F), has increased the tropism and transduction success of AAV vectors for the inner retina [129,135,136]. 

### 4.2. Non-Viral Gene Delivery Strategies

Two non-viral gene delivery strategies that have also been explored in preclinical work include electroporation and nanoparticles, and while these technologies have not yet been translated to clinical trials, they have demonstrated success in preclinical models of IRD. Electroporation involves the injection of DNA into the subretinal space followed by the delivery of a high voltage pulse that drives the DNA into the cell through changes in the cellular membrane potential and permeability [137,138]. Nanoparticles, which include a broad range of materials such as liposomes, polymers, and peptide-coupled DNA, have been studied in the context of ocular gene therapy [106]. While they have lower transfection efficiency than AAV, their development might be more cost-effective, allow for larger genetic payload (up to 20 kb), and is suggested to lead to a lower likelihood of genomic integration and tumorigenicity [139,140,141,142,143]. 

## 5. RNA-Modifying Therapies for Inherited Retinal Degenerations

While the previously mentioned gene augmentation strategies are useful for retinal degenerations caused by loss-of-function mutations or dominant haploinsufficiency, they are not appropriate to address dominant mutations resulting from gain-of-function or dominant-negative alleles. One important strategy to modulate the expression of these detrimental alleles relies on degrading messenger RNA (mRNA) before it can be translated to protein. Within this category, RNA interference (RNAi) perhaps has the most extensive history of investigation as a potential therapy for IRDs. RNAi works by post-transcriptional gene silencing of messenger RNA (mRNA). These technologies include: ribozymes, short-interfering RNA (siRNA), short-hairpin RNA (shRNA), and antisense oligonucleotides (AONs) [144]. Ribozymes are naturally-occurring RNAs in the RNase-P complex that convert precursor tRNA to its active form. Ribozymes can be engineered in the laboratory to cleave specific mRNAs to prevent their translation into protein, but low in vivo catalytic activity has limited the applicability of ribozymes for the treatment of IRDs [145]; however, enhancements in kinetic activity continue to be made to develop these for clinical applications [146,147]. siRNA and shRNA are double-stranded RNAs that when expressed in a cell are processed to single-strand antisense guides that are incorporated into the RNA-induced silencing complex (RISC) and degrade complementary mRNA [148]. siRNA can be encapsulated in lipid delivery vehicles, and shRNA are packaged in viral vectors. shRNA can also be delivered alongside a wild-type coding sequence for the gene of interest for which the codon usage has been modified to make it resistant to the RNAi strategy and make it suitable for “knockdown-and-replace” strategies for autosomal dominant disease, such as in dominant RHO-associated RP [149,150]. This led to the development of a dual AAV vector therapy RhoNova (Roche), but there has been no update on its clinical development at the time of this publication. More recently, a single AAV2/5 vector expressing both the shRNA and a normal copy of the RHO gene has been developed [151], and it is currently in development for a Phase 1/2 clinical trial with IVERIC bio. 

Other novel RNA-based therapies incorporate the use of antisense oligonucleotides (AONs or ASOs), which are synthetic single-stranded RNA or DNA that bind complementary mRNA transcripts. AONs can modify gene expression by multiple mechanisms including inhibition of translation, cleavage and degradation of mRNA transcripts, or alteration of pre-mRNA splicing leading to splice site inclusion or exclusion in frameshift alleles that would otherwise lead to an early stop or transcript degeneration [152]. These nucleic acid fragments can be delivered to cells in the retina by intravitreal administration without the need for viral or even lipid vectors, making them attractive for the treatment of IRDs. While AONs can be likely delivered less invasively, they will likely require repeat injections over the lifetime of the individual. 

Currently, Phase 2/3 trials sponsored by ProQR Therapeutics (NCT03913143, ILLUMINATE, and NCT04855045, BRIGHTEN) are testing the efficacy of an AONs-based therapy, sepofarsen, for the treatment of the most common mutation in the most frequently causative gene for LCA, CEP290 [153,154] (Table 1). This allele is characterized by a point mutation (p.Cys998X, or IVS26) that generates a cryptic splice donor site that leads to an introduction of an early stop. The results of the Phase 1/2 study (NCT03140969) were published in 2019, and the data from ten out of the eleven patients that received this therapy suggested clinically meaningful improvement in visual acuity with treated eyes measuring 0.54 log10 MAR (26 letters) better than untreated eyes 3 months after the initial dose [154]. Treated eyes also demonstrated better performance in full-field stimulus testing (FST) for blue light, when compared to untreated eyes [154]. CEP290 is a transition zone protein involved in regulation of the normal traffic of proteins from the inner segments to the outer segments, and the surviving cone photoreceptors in patients with this condition are usually morphologically abnormal [155,156,157]. Treatment with sepofarsen appeared to improve imaging correlates of normal photoreceptor anatomy at the inner to outer segment junction in two patients [154]. At three months, ten out of the eleven subjects received a second injection. The eleventh patient in the trial refused repeated injections to avoid early cataracts, and recently, a case report was published reporting improvements in the treated eye of visual acuity, FST light sensitivity, mobility testing, and pupil constriction latency after one single injection. The maximum benefit was reported at approximately two months, but he still demonstrated sustained benefit for at least fifteen months [153]. Interestingly, the authors reported a transient increase in OCT reflectivity near the photoreceptor ciliary transition zone between the third and fifth month, which correlated with the patient’s improvement in visual function. They suggest that this finding could represent an imaging correlate between improvements in structure and function as a result of the treatment [153]. ProQR Therapeutics announced the completion of enrollment for their Phase 2/3 trial (Illuminate) and anticipates top-line results will become available in the first half of 2022. 

AONs are now being investigated for the treatment of RP caused by mutations in exon 13 of USH2A (NCT03780257: STELLAR, Sponsor: ProQR Therapeutics) and dominant RHO(P23H) (NCT04123626: AURORA, Sponsor: ProQR Therapeutics) (Table 1). Variants in USH2A and RHO are the most frequent causes of autosomal recessive and autosomal dominant RP, respectively. 

## 6. Gene Editing Technologies

### 6.1. CRISPR-Cas9 Genome Editing

Recent innovations in gene-editing technologies have now extended the mechanisms of gene therapy beyond replacement of loss-of-function mutations or repression of a mutant allele but allow the precise editing of the genome itself to allow the expression of a normal protein. Most gene-editing strategies rely on inducing DNA breaks that are repaired by the natural DNA-repair machinery. While preclinical studies have identified multiple enzymes that can be used to cut DNA specifically at locations of interest including transcription activator-like effector nucleases (TALENs) and zinc-finger endonucleases (ZFNs) [158], most current gene- editing technologies in development are leveraging the bacterial enzyme Cas9 for therapeutic gene editing. The first in vivo gene editing trial in humans used a ZFN to treat mucopolysaccharidosis II (MPS II) or Hunter’s syndrome (Sangamo, NCT03041324) [159]. 

While the former two technologies require a new protein to be designed for each genomic targeting site, the CRISPR-Cas9 system can be modified to target a specific genomic locus by changing the sequences of the guide RNA with traditional molecular cloning techniques [160]. The bacterial CRISPR-Cas9 system is based on a DNA endonuclease known as Cas9, which can be targeted to DNA areas of interest using a customizable RNA known as the single-guide RNA (sgRNA). CRISPR is an acronym for “clustered regularly interspaced short palindromic repeats” for the specific DNA sequences naturally present in the bacterial and archaeal genomes that the Cas9 enzyme recognizes to mediate its effect against viral sequences. The revolutionary research and therapeutic potential of this new technology culminated in Emmanuelle Charpentier and Jennifer Doudna receiving the 2020 Nobel Prize in Chemistry for their pioneering work in CRISPR-Cas9 technology. Some of the other reasons for its wide popularity include a greater ease in cloning viral vectors for delivery to cells, and the ability to target multiple genes simultaneously if desired by co-expressing multiple guide RNAs [161]. 

Since double-stranded breaks in DNA can lead to the initiation of apoptosis (programed cell death) if not corrected, eukaryotic cells have developed multiple mechanisms to rapidly fix the double-stranded breaks. The most frequently invoked mechanism is called non-homologous end joining (NHEJ) [162], which is a low-fidelity mechanism that joins together the two broken ends of the DNA sequence, and often leads to short insertions or deletions that often disrupt the targeted gene product through frameshift mutations that can often lead to an early stop codon and loss of expression through a mechanism termed nonsense mediated decay [163]. This strategy has been explored for the treatment of IRDs caused by dominant negative and gain-of-function alleles, such as the RHO(S334) allele [164], where knocking-out the expression of the entire mutated allele would be desirable. 

An alternative mechanism known as homology-directed repair (HDR), uses the homologous chromosome or a homologous DNA sequence as a template to guide the repair of the broken DNA strand to ensure high-fidelity repair prior to “gluing” the two ends together [165]. While HDR would be an ideal method for gene-editing technologies because it would allow for a wild-type copy of the gene to replace the mutant allele, this strategy is limited by the low efficiency rate of HDR in somatic cells [166]. Therefore, a gene-editing strategy that relies on this method might only correct the mutant allele on a small fraction of cells, and this might not provide a significant improvement in vision for the patient. 

### 6.2. Advanced Gene-Editing Technologies 

Base editing is the first of these new technologies that aimed to shift the efficiency from imperfect end-joining to precise gene editing [167,168]. It uses a modified Cas9 engineered to produce only single-stranded breaks in the DNA and an enzyme to switch one specific DNA base for another near the nick site. This enzyme is a nucleoside deaminase—either a naturally occurring cytidine deaminase [168] or an engineered adenosine deaminase [167]—which is selected for the desired mutation, but it can only make four types of base transitions: C-to-T, G-to-A, A-to-G, or T-to-C [169]. Nevertheless, transition mutations represent in aggregate approximately 30% of all human pathogenic variants [14]. While this was a milestone advance in precision gene editing, it did not provide solutions for insertions, deletions, frameshift mutations, or the flexibility to cover all base pair transitions and transversions [169].Prime editing [170] is the most recent adaptation of the CRISPR technology and provides even more versatility than base editing. This new technology is based on a modified Cas9 endonuclease that only induces single-stranded breaks, and a reverse transcriptase enzyme that can generate new customizable DNA from the prime editing RNA guide at the cut site. This system requires a two-part RNA guide. The ‘search’ part of the guide directs Cas9 to a specific sequence in the DNA target, where it causes a single-stranded DNA break. Then, the reverse transcriptase produces a DNA complementary to the sequence in the ‘replace’ part of the RNA guide and incorporates it on one of the cut DNA ends, replacing the original sequence [170]. This technology can be applied to correct point mutations including all four transition point mutations, and all eight transversion point mutations. Furthermore, it can also be used to edit the genome through small insertions (1 bp to 44 bp) or deletions (1 bp to 80 bp) or combinations of the above [170], providing unprecedented flexibility and precision of genomic manipulation. However, while prime editing increases the efficiency of templated DNA repair compared to prior gene editing technologies, imperfect DNA repair can still occur. Furthermore, the machinery required for this system is twice as large as that in the conventional Cas9 system, and it is possible that gene delivery to some cell types might be challenging [171]. Another limitation is the relatively small size of the insertions that can be reliably achieved through this technology, which is possibly attributable to the constraints imposed by the stability of the pegRNA that is used as the template to make the homologous repair. While this new method has the potential to increase the flexibility of templated gene -editing, more work is still needed before prime editing can be applied in medical settings, where unintended gene rearrangements would be unacceptable. 

### 6.3. Gene Editing and the Treatment of Retinal Disease

The first CRISPR-Cas9- based gene editing trial in humans is targeting an inherited retinal dystrophy and is currently underway. BRILLIANCE (NCT03872479) is a Phase 1/2 study sponsored by Editas Medicine (Cambridge, MA, USA) using CRISPR-Cas9 gene editing to treat the most common disease-causing variant responsible for LCA, CEP290 c.2991 + 1655A > G (IVS26) [172] (Table 1). As previously mentioned, IVS26 introduces a cryptic splice donor site resulting in the inclusion of a pseudoexon that leads to the premature truncation of the CEP290 protein and dysfunction [120]. Given the size of the CEP290 coding sequence, this allele cannot be treated through traditional gene augmentation within an AAV vector carrying the full coding sequence. In the trial, an AAV5-based vector uses the GRK1 promoter to achieve photoreceptor-specific expression of Cas9 and two CEP290-specific sgRNAs. While clinical data from this trial have yet to be released, preclinical data from experiments performed in transgenic mice expressing human CEP290 (IVS26) and non-human primates demonstrated that this strategy was successful in effective gene editing and recovery of expression of the full-length CEP290 protein in mature photoreceptors [120]. Antibodies directed against AAV5 were detected in the sera of non-human primates, but they could not detect an immune response directed at Cas9 [120]. While more clinical data are required to determine the safety and efficacy in humans of this approach, the preclinical studies were encouraging at reaching the goal of editing the genome of greater than 10% of foveal cones [120]. Achieving this percentage of editing is meaningful: prior studies of patients with Stargardt disease estimated that they possessed 1/10th of the normal foveal cone density, but still retained near normal vision until the 5th decade of life [120,173]. Editas Medicine has reported that while the efficiency of gene-editing is dependent on the titer of the virus [120], the titer under clinical investigation is comparable to the one used for commercial voretigene neparvovec-rzyl (Luxturna) [174]. 

## 7. Restoring Light Sensitivity to the Retina with Optogenetics 

In many patients with later-stage retinal degeneration where many, if not all, of the photoreceptors have degenerated, the inner layers remain populated with interneurons and RGCs. These remain connected to targets in the optic tectum and visual cortex. These surviving cells have been shown to remain functional despite the structural changes that occur following photoreceptor loss [175,176,177]. Optogenetic therapy aims to confer light sensitivity to these surviving retinal neurons by expressing a microbial photopigment, such as channel rhodopsin (ChR2) [178]. Microbial photopigments are light-sensitive cation or anion channels that have the ability to depolarize or hyperpolarize a cell in response to light in a specific wavelength [179]. 

Optogenetic strategies are currently being explored in two active human clinical trials. Retrosense Therapeutics, now a part of Allergan, is conducting a Phase 1/2 clinical trial (NCT02556736) delivering ChR2 with an AAV2 viral vector via intravitreal injections into patients with advanced retinitis pigmentosa (RP) (Table 1). The trial was initiated in 2015, and results have not been yet published. The PIONEER trial, sponsored by GenSight Biologics (NCT03326336), also involves the intravitreal delivery of an AAV2(7m8) vector carrying a different photopigment, the ChrimsonR-tdTomato fusion protein [180]. The therapy also involves the use custom-designed goggles to project stimulating light pulses to the patient’s retina in response to changes in environmental light. Recently, data were published from a single patient with RP participating in this trial (PIONEER) supporting the safety and efficacy of ChrimsonR expression paired with light-stimulating goggles in restoring some evidence of visual function [181]. While these data are encouraging, it is hard to extrapolate on effectiveness based on a single patient. Adding to the possible limitations of this technology, they report that the region of optogenetic expression likely corresponds to a 2.5 mm diameter disk of retina [181]. 

In theory, this strategy has the potential to become a universally applicable therapy for retinal degenerations regardless of the causative mutation if sufficient RGCs persist. In some IRDs, rod photoreceptors are lost before cone photoreceptors, suggesting that the surviving cone photoreceptors could be targeted by optogenetic interventions designed to improve light sensitivity and vision [182,183]. Depending on the disease, different surviving cell populations might need to be targeted. Some models suggest that targeting cells more proximal in the visual pathway such as cones or ON-bipolar cells would provide the highest visual potential [179]. In a healthy retina, a light stimulus results in the depolarization of ON-bipolar cells and the hyperpolarization of OFF-bipolar cells. Then, the bipolar cells relay this information to amacrine cells and RGCs, whose axons terminate in the lateral geniculate nucleus of the thalamus and relay the visual input to the brain. Targeting the visual pathway earlier in this network may provide more opportunity to make use of endogenous visual processing networks; for example, targeting bipolar cells rather than RGCs. However, in some instances, targeting RGCs directly might provide useful vision for patients when significant numbers of bipolar cells have degenerated [179]. 

There are several limitations to the optogenetic approach that remain to be addressed. First, the currently available microbial opsins have lower light sensitivity than rhodopsin or other vertebrate opsins. In fact, initial studies using ChR2 demonstrated that the light required to activate some of the transformed retinal cells would be much higher than that encountered in normal lighting conditions [184,185]. The human retina can be damaged by certain light intensities and wavelengths [186,187], and safety thresholds for humans have been established [179]. The initial studies for ChR2 in mammalian retina required stimulation with blue light at high intensities that were concerning for long-term phototoxicity given that they exceeded the established safety thresholds for artificial radiation to the human retina [188]. This inspired the development of red-shifted opsins, such as red-shifted channelrhodopsin (ReaChr) and ChrimsonR, which have been shown to be activated by orange light at safe intensities [188]. Continuous light exposure at very high intensities combined with the expression of a modified Volvox channelrhodopsin-1 (mVChR1) in rat retina [189], which responds to a broad range of light wavelengths in the visual spectrum, did not cause RGC degeneration [190]. The opsins being investigated in optogenetic studies are microbial proteins from bacteria, algae, or fungi, and the safety and potential immunogenicity of these constructs remains to be understood once these elements are expressed in the human eye. Furthermore, there remain technical challenges for the efficient transformation of inner retinal cells with the use of AAV vectors. Experiments performed in -vivo and ex -vivo in non-human primates have demonstrated limited transduction of RGCs and intra-ocular inflammatory responses seen in treated subjects [191,192,193]. Current clinical trials have shown that AAV8 and AAV2 vectors are effective at targeting photoreceptors and cells in the RPE [110,194], with the AAV8(BP2) variant [195] being an attractive option for targeting the inner retina. New AAV variants that have high transduction affinity for ON-bipolar cells and cell-specific promoters would be desirable for the future application of optogenetic therapies [179]. 

While the previously discussed trials are using microbial photopigments, Kubota Vision is currently developing an optogenic approach based on human rhodopsin targeting ON-bipolar cells. One key distinction for this approach is that unlike microbial opsins that act as a light-sensitive channel, rhodopsin requires additional molecules for phototransduction that are not normally present in ON-bipolar cells; however, the company has not released further details regarding this treatment. Advantages may include using a human photopigment, which may decrease the risk of immunogenicity, and the fact that rhodopsin is much more sensitive to levels of illumination below that of currently available bacterial opsins.

## 8. Retinal Cell Replacement Therapies 

The introduction of induced pluripotent stem cells (iPSCs) into the visual sciences has provided an outstanding resource for the modeling of human retinal degenerations in the laboratory, and it also generated hope for future therapies capable of replacing the lost cells as a consequence of retinal dystrophies. iPSC technology has allowed the generation of human photoreceptors and retinal cells in culture from patients with disease from adult somatic biopsies (e.g., blood and skin samples). These human stem cell cultures carry the disease-causing variant, and methods to differentiate retinal cells can serve as a model for understanding the mechanism of IRDs. These have become increasingly promising for the many cases in which there is no suitable animal model for a particular human mutation [196]. iPSCs have also provided a platform for proof-of-concept experiments of novel therapeutics, including the viral vector currently being used for a Phase 1/2 clinical trial for the treatment of choroideremia [39]. 

### 8.1. Stem Cell- Derived Photoreceptor Cells 

Pre-clinical work has demonstrated that we can successfully grow photoreceptor cells in human retinal organoids in numbers that could potentially be used for cell replacement in a variety of retinal diseases [42,197]. Human organoids are three-dimensional, self-organizing, structures that are grown in culture from adult, embryonic, or induced pluripotent stem cells. Retinal organoids recapitulate the laminated ultrastructure of the retina, as well as the diversity and spatial organization of primary retinal cell types within these layers. Experiments with these organoids in vitro have shown some limited responses to light that can be detected from downstream RGCs [198]. The limited sensitivity to light of organoids has been attributed to poor photoreceptor outer segment development, which might be secondary to the limited interaction between photoreceptors and the patchy RPE layer present in many of the current retinal organoids [197,198]. Recently, retinal organoids with three nuclear and two synaptic layers from human iPSCs were successfully grown that possess the ability to respond to light and transmit the light responses to second-order or third-order retinal cells as measured by changes in calcium transients with the use of the genetically encoded calcium indicator GCaMP6s [199]. Photosensitive activity was also described with the use of electroretinography (ERG) recordings in bilaterally symmetric optic vesicle-like structures that self-organized from human brain organoids derived from iPSCs [200]. These optic-vesicle-like structures included retinal progenitor cells, retinal pigment epithelia, axon-like projections, corneal epithelial cells, and lens-like cells [200]. As it continues developing, this technology will continue to offer more opportunities to study the pathophysiology of retinal dystrophies and, test new potential therapies in human cells, and it could possibly lead to the generation of whole grafts of functional retinal tissue that could be transplanted to patients [197]. Here, we will discuss the application of these technologies for replacing photoreceptors and RPE cells. Similar efforts for the transplantation of RGCs are currently underway, and these approaches hold promise for the future treatment of glaucoma and optic neuropathies, which are reviewed elsewhere [42,201,202,203,204]. 

Over the past 15 years, multiple groups have demonstrated progress in pre-clinical models with the transplantation of rod photoreceptor precursors derived from stem cells into mouse models [205,206]. These preclinical studies have suggested that these transplanted cells can restore elements of visual function [207,208,209,210]. Initially, it was proposed that some of these photoreceptors, which were molecularly labeled with green fluorescent protein (GFP), were successful at integrating to the host retina and partially restoring vision [211]. The morphology of these cells was interpreted as evidence of integration of donor cells. In the last four years, we have seen a major shift in our understanding of donor cell “engraftment” into adult retina: in many cases, donor cells exchange RNA and protein with host photoreceptors through a mechanism known as material transfer (MT) [212,213,214,215,216]. A re-analysis of previous experiments with photoreceptor cell transplantation into adult mouse retina showed that MT between donor and host photoreceptors was responsible for the presence of GFP-labeled cells in the outer nuclear layer of recipient animals. Recently, multiple groups have proposed that the transplanted photoreceptor precursor cells may be able to provide support material for the surviving host photoreceptors, in addition to generating new photoreceptor cells that incorporate into the retina. These groups hypothesize that the extent to which MT versus true cellular integration occurs is, in part, determined by the host retinal environment. In healthy, wild-type retinas, material transfer appears to be the dominant process, while in models of retinal degeneration that have significant host-photoreceptor loss, precursor incorporation may be the dominant process [217]. For example, a recent study reported the successful transplantation of sorted hPSC-derived cones into the rd1 mouse model of retinal degeneration. These human cones formed functional connections with murine bipolar cells, restoring photopic light-evoked retinal ganglion cell responses and light-evoked behaviors in the treated animals [218]. While these reports are encouraging, further studies are needed to demonstrate whether these transplanted neurons can form new synapses to the host retina in human tissue and result in measurable improvements in vision. 

ReNeuron, a UK-based company, is testing the effects of hRPCs delivered with a subretinal injection as treatment for RP (clinicaltrials.gov identifier NCT02464436) [42]. Preclinical studies suggested that when transplanted into the retina, hRPCs not only helped to preserve existing photoreceptors, but some progenitors matured into functional photoreceptors that engrafted into the host retina [219,220]. In early July, 2021, ReNeuron reported an encouraging efficacy signal in Phase 2a subjects with some variability among patients and announced regulatory approval for a 3-month Phase 2a extension study in the US, UK, and Spain that incorporates a higher therapeutic cell dose. 

### 8.2. Retinal Pigmented Epithelium (RPE) Cells 

While many retinal dystrophies are caused by mutations that directly affect photoreceptor function, there are both inherited and acquired retinal degenerations that are characterized by defects in RPE function. The RPE is a monolayer of polarized columnar epithelial cells on an organized basement membrane (Bruch’s membrane), with an apical surface in contact with the outer segments of photoreceptors. The RPE plays critical functions ensuring the proper function of photoreceptors including phagocytosis of the shed outer segment discs, regeneration of 11-cis-retinal during the visual cycle, and the secretion of growth factors [221]. Beyond providing support to the photoreceptors, the RPE also supports the overall function of the retina by maintaining the outer blood-retina barrier via intercellular tight junctions [221]. 

RPE transplantation was initially proposed nearly 30 years ago for the treatment of exudative AMD, when it became clear that removal of the choroidal neovascular membranes was not sufficient to improve visual outcomes in several studies, including the Submacular Surgery Trials [222]. Then, it was hypothesized that the compromised RPE had to be replaced with healthy RPE to achieve visual improvement. This realization led to multiple small studies that transplanted RPE from various sources, including fetal RPE cells, autologous cells, and the developmentally related iris pigment epithelium cells [221,223,224]. Other early approaches for the treatment of exudative AMD included the surgical transplantation of an autologous graft of RPE, Bruch’s membrane, choriocapillaris, and choroid from the retinal periphery to the macula to treat the remaining areas of RPE atrophy after the surgical removal of sub-macular scar tissue [225,226]. This procedure was shown to be successful in a small subset of patients, resulting in stable BCVA for up to seven years [227]. However, these approaches did not gain widespread support given the lack of conclusive data on visual improvement and associated complications including proliferative vitreoretinopathy, macular pucker, or recurrence of CNV [221,225]. Nevertheless, these studies strongly suggested that transplanted RPE cells could be developed as potential treatment for AMD [42,221]. 

More recently, functional RPE cells have been successfully derived from various types of stem cells, including human embryonic stem cells (hESCs), inducible pluripotent stem cells, and a population of human RPE stem cells [42]. Two different approaches are being tested for transplantation of these stem cell-derived RPE cells: injection of an RPE cell suspension, or the transplantation of an RPE monolayer patch. In a Phase 1/2a trial, an ESC-derived RPE cell suspension was delivered to patients with non-exudative AMD (geographic atrophy) and Stargardt disease. After a two-year follow-up period, the investigators reported no serious adverse effects arising from the procedure [228,229]; however, there were some concerns that the injected suspension of RPE cells did not reliably form a monolayer of RPE cells and had poor long-term survival when compared to cells transplanted as a monolayer patch [230,231]. 

The concept of transplanting RPE already as a monolayer patch aims to recapitulate the normal anatomy of the RPE and foster the formation of tight junctions between neighboring cells. These contacts are important in the establishment of cell polarity that is necessary for the RPE cells to gain the ability to perform their normal support functions for the retina and photoreceptors [232]. By growing a functional RPE monolayer patch ex vivo, researchers can perform RPE functionality readouts prior to surgical implantation of the graft, which is something that is not possible to do with RPE cell suspensions [233,234,235]. Data on three different types of RPE patches from independent studies including patients with exudative and non-exudative AMD have been published, and reports suggest visual stability or moderate improvement in a very small number of patients [235,236,237]. While encouraging, these studies are limited by the small number of patients and short follow-up period. 

## 9. Conclusions 

Innovations in molecular biology, stem cell biology, and visual science have continued to expand our toolkit for treating vision loss as a result of retinal dystrophies. Many of these new approaches are actively being tested in clinical trials and will hopefully result in effective therapies that can either prevent further vision loss or restore vision in a growing number of patients. Inspired by the success of gene augmentation strategies, many of the actively researched therapies hold the promise of offering therapies that will span the full spectrum of causative mutations behind IRDs. With the application of new technologies, including the first gene-editing trial using CRISPR-Cas9 for the treatment of CEP290 disease actively underway, we will soon learn about the safety and efficacy of this promising approach in humans. Innovations in the effectiveness and safety of gene-editing approaches will hopefully increase the variety of conditions that could be treated and minimize concerns about off-target genetic effects. Other exciting possibilities include the success of neuroprotection, optogenetics, or regenerative cell-based therapies that are theoretically mutation -agnostic. Some of these approaches could offer the possibility of restoring vision to patients with later-stage disease or other acquired retinal degenerations. The success of any of these approaches would have a lasting impact on the future practice of ophthalmology and medicine. 

## Figures and Tables

**Table 1:** Recent clinical trials for retinal dystrophies arranged by condition and therapeutic approach [21,22,23,24,25,26,27,28,29,30,31,32,33,34,35,36,37].

Condition	Phase	Vector/Drug	Gene	Prevalence	Allele Mechanism	Sponsor	Status
**Adeno-Associated Virus Vector Gene Therapies**							
Achromatopsia	1/2	AAV2tYF	*CNGB3*	50% [21]	LOF	Applied Genetic Technologies Corp	Recruiting
	1/2	AAV2/8	*CNGB3*	“	“	MeiraGTx	Completed
	1/2	AAV2tYF	*CNGA3*	25% [21]	LOF	Applied Genetic Technologies Corp	Recruiting
	1/2	AAV2/8	*CNGA3*	“	“	MeiraGTx	Recruiting
	1/2	AAV8	*CNGA3*	“	“	STZ Eyetrial	Recruiting
X-Linked Retinoschisis	1/2	AAV2tYF	*RS1*	100% [22]	LOF	Applied Genetic Technologies Corp	Recruiting
	1/2	AAV8	*RS1*	“	“	National Institutes of Health	Recruiting
X-Linked RP	1/2	AAV2/5	*RPGR*	75% [23]	LOF	MeiraGTx	Recruiting
	1/2	AAV(4D-R100)	*RPGR*	“	“	4D Molecular Therapeutics	Recruiting
	1/2 > 2	AAV2tYF	*RPGR*	“	“	Applied Genetic Technologies Corp	Recruiting
	2 > 3	AAV2/8	*RPGR*	“	“	Biogen/NightstaRx	Recruiting
Autosomal Recessive RP (arRP)	1/2	AAV2/5	*PDE6B*	4% [24]	LOF	Horama S.A.	Recruiting
	1/2	AAV8	*RLBP1*	1% [25]	“	Novartis	Recruiting
	1/2	AAV2	*MERTK*	4% [26]	“	King Khaled Eye Specialist Hospital	Completed
Choroideremia	1	AAV2	*REP1*	100% [27]	LOF	4D Molecular Therapeutics	Recruiting
	1/2	AAV2	*REP1*	“	“	Spark Therapeutics	Active, Not Recruiting
	2	AAV2	*REP1*	“	“	STZ Eyetrial	Completed
	2	AAV2	*REP1*	“	“	University of Oxford	Completed
	2	AAV2	*REP1*	“	“	Bascom Palmer/University of Miami	Completed
	1/2	AAV2	*REP1*	“	“	University of Alberta	Completed
	3	AAV2	*REP1*	“	“	Biogen/NightstaRx	Completed
LCA2/arRP	Approved	AAV2	*RPE65*	6% LCA [28], 2% arRP [29]	LOF	Spark Therapeutics	Treating
LCA1	1/2	AAV5	*GUCY2D*	10–20% [30]	LOF	Atsena Therapeutics	Recruiting
**Lentivirus Vector Gene Therapies**							
Stargardt Disease	1/2	EIAV	*ABCA4*	100% [31]	LOF	Sanofi	Terminated
Usher Syndrome 1B	1/2	EIAV	*MYO7A*	21% [32]	LOF	Sanofi	Terminated
**Antisense Oligonucleotides**							
Usher Syndrome 2/ arRP	1/2	QR-421a	*USH2A (exon 13)*	~20% US2, 4% arRP [33,34,35] ††	frame-shift/LOF	ProQR Therapeutics	Recruiting
RP	1/2	QR-1123	*RHO(P23H)*	10% [36] ††	missense/dominant negative	ProQR Therapeutics	Recruiting
LCA10	1/2	Sepofarsen (QR-110)	*CEP290(p.Cys998X)*	10–15% of LCA [37] ††	splice-donor site/insertion	ProQR Therapeutics	Recruiting
**Gene editing/ CRISPR Cas9**							
LCA10	1/2	EDIT-101	*CEP290(p.Cys998X)*	10–15% of LCA [37] ††	splice-donor site/insertion	Editas Medicine	Recruiting
**Optogenetics**							
RP	1/2	AAV2-7m8-ChrimsonR-tdTomato	***			GenSight Biologics	Recruiting
	1/2	AAV2-ChR2	***			Allergan	Active, Not Recruiting
	2	AAV2-MCO-010	***			Nanoscope Therapeutics	Recruiting
**Antioxidants and neuroprotection**							
RP	1	*N-acetylcysteine*	***			Johns Hopkins University	Active, Not Recruiting
Usher Syndromes	1/2	NPI-001 tablets	***			Nacuity Pharmaceuticals Foundation Finding Blindness	Recruiting
Stargardt Disease	2	ALK-001	*ABCA4*	100% [31]	LOF	Alkeus Pharmaceuticals	Recruiting
	2	STG-001	“	“	“	Stargazer Pharmaceutical	Completed
	2	avacincaptad pegol (Zimura)	“	“	“	IVERIC Bio	Recruiting
	3	Emixustat	“	“	“	Kubota Vision	Active, Not Recruiting
**Stem cells and cell-based therapies**							
RP	1	intravitreal CD34+ Stem Cells	***			University of California, Davis Cures within Reach	Recruiting
	1/2	subretinal hRPCs	***			ReNeuron Limited	Recruiting
	2	intravitreal hRPCs	***			jCyte	Active, Not Recruiting
Stargardt Disease	1/2	Subretinal injection of human embryonic stem cell-derived RPE cells				Astellas Institute for Regenerative Medicine	Completed

The following abbreviations were used in this table: RP—rRetinitis pPigmentosa, arRP; autosomal recessive rRetinitis pPigmentosa; LCA—Leber congenital amaurosis; US2—Usher syndrome type 2; AAV—aAdenoassociated virus; EIAV—eEquine infectious anemia virus; ChR2—channelrhodopsin 2; hRPCs—human retinal progenitor cells; RPE—retinal pigmented epithelium; LOF—loss-of-function. *** Genotype not specified by trial; †† Reported prevalence is for the listed allele “ Same as previous entry.

## Data Availability

Not applicable as this is a review article.
